# Persistence of Supplemented *Bifidobacterium longum* subsp. *infantis* EVC001 in Breastfed Infants

**DOI:** 10.1128/mSphere.00501-17

**Published:** 2017-12-06

**Authors:** Steven A. Frese, Andra A. Hutton, Lindsey N. Contreras, Claire A. Shaw, Michelle C. Palumbo, Giorgio Casaburi, Gege Xu, Jasmine C. C. Davis, Carlito B. Lebrilla, Bethany M. Henrick, Samara L. Freeman, Daniela Barile, J. Bruce German, David A. Mills, Jennifer T. Smilowitz, Mark A. Underwood

**Affiliations:** a Evolve Biosystems, Inc., Davis, California, USA; b Department of Chemistry, University of California, Davis, California, USA; c Foods for Health Institute, University of California, Davis, California, USA; d Department of Food Science and Technology, University of California, Davis, California, USA; e Department of Viticulture and Enology, University of California, Davis, California, USA; f Department of Pediatrics, UC Davis Children’s Hospital, Sacramento, California, USA; Arizona State University

**Keywords:** bifidobacteria, breast milk, human milk oligosaccharides, infant microbiome

## Abstract

Attempts to alter intestinal dysbiosis via administration of probiotics have consistently shown that colonization with the administered microbes is transient. This study sought to determine whether provision of an initial course of *Bifidobacterium longum* subsp. *infantis* (*B. infantis*) would lead to persistent colonization of the probiotic organism in breastfed infants. Mothers intending to breastfeed were recruited and provided with lactation support. One group of mothers fed *B. infantis* EVC001 to their infants from day 7 to day 28 of life (*n* = 34), and the second group did not administer any probiotic (*n* = 32). Fecal samples were collected during the first 60 postnatal days in both groups. Fecal samples were assessed by 16S rRNA gene sequencing, quantitative PCR, mass spectrometry, and endotoxin measurement. *B. infantis*-fed infants had significantly higher populations of fecal *Bifidobacteriaceae*, in particular *B. infantis*, while EVC001 was fed, and this difference persisted more than 30 days after EVC001 supplementation ceased. Fecal milk oligosaccharides were significantly lower in *B. infantis* EVC001-fed infants, demonstrating higher consumption of human milk oligosaccharides by *B. infantis* EVC001. Concentrations of acetate and lactate were significantly higher and fecal pH was significantly lower in infants fed EVC001, demonstrating alterations in intestinal fermentation. Infants colonized by *Bifidobacteriaceae* at high levels had 4-fold-lower fecal endotoxin levels, consistent with observed lower levels of Gram-negative *Proteobacteria* and *Bacteroidetes*.

**IMPORTANCE** The gut microbiome in early life plays an important role for long-term health and is shaped in large part by diet. Probiotics may contribute to improvements in health, but they have not been shown to alter the community composition of the gut microbiome. Here, we found that breastfed infants could be stably colonized at high levels by provision of *B. infantis* EVC001, with significant changes to the overall microbiome composition persisting more than a month later, whether the infants were born vaginally or by caesarean section. This observation is consistent with previous studies demonstrating the capacity of this subspecies to utilize human milk glycans as a nutrient and underscores the importance of pairing a probiotic organism with a specific substrate. Colonization by *B. infantis* EVC001 resulted in significant changes to fecal microbiome composition and was associated with improvements in fecal biochemistry. The combination of human milk and an infant-associated *Bifidobacterium* sp. shows, for the first time, that durable changes to the human gut microbiome are possible and are associated with improved gut function.

## Abstract (precis)

The gut microbiome in early life plays an important role for long-term health and is shaped in large part by diet. Probiotics may contribute to improvements in health, but they have not been shown to alter the community composition of the gut microbiome. Here, we found that breastfed infants could be stably colonized at high levels by provision of *B. infantis* EVC001, with significant changes to the overall microbiome composition persisting more than a month later, whether the infants were born vaginally or by caesarean section. This observation is consistent with previous studies demonstrating the capacity of this subspecies to utilize human milk glycans as a nutrient and underscores the importance of pairing a probiotic organism with a specific substrate. Colonization by *B. infantis* EVC001 resulted in significant changes to fecal microbiome composition and was associated with improvements in fecal biochemistry. The combination of human milk and an infant-associated *Bifidobacterium* sp. shows, for the first time, that durable changes to the human gut microbiome are possible and are associated with improved gut function.

## INTRODUCTION

In many resource-poor countries, bifidobacteria are the dominant fecal microbes in breastfed infants (1–3), whereas in resource-rich countries there is marked variability, with some studies showing low numbers of fecal bifidobacteria among breastfed infants (4–6). Infant delivery mode, diet, and maternal fecal bifidobacteria influence infant colonization with bifidobacteria. Decreased numbers of intestinal bifidobacteria have clinical relevance, based on a large body of evidence that intestinal dysbiosis early in life predisposes to inflammation, and increases risks for obesity, atopic and allergic diseases, inflammatory bowel disease (7–9), and diabetes mellitus (types 1 and 2) (6, 10). It is not likely coincidental that dysbiosis-associated diseases are markedly less common in resource-poor countries.

Bifidobacteria appear to be particularly important, given the evidence for reversal of stress-induced inflammation and intestinal hyperpermeability both *in vitro* and in animal models (11–13). Probiotics hold promise as a potential modality to correct dysbiosis in early life and prevent gut microbiota-associated diseases; however, many commercially available probiotic products have shown only limited benefits to date. This may be due in part to the high degree of variability in purity and viability of current probiotic products (14). In addition, probiotic trials to date have demonstrated only transient colonization with the administered strain (15–17). This is likely a consequence of the ecological and evolutionary adaptations of the probiotic strains used, as stable persistence of an exogenous strain is possible (18).

Human milk contains prebiotic oligosaccharides that are not digestible by the host infant but are rapidly consumed by a limited number of species of *Bifidobacterium* and *Bacteroides* (19). *Bifidobacterium longum* subsp. *infantis* (*B. infantis*) is unique among gut microbes in its capacity to transport into its cytoplasm and consume the full range of human milk oligosaccharides (HMOs) (20). We previously reported a clinical trial of *B. infantis* EVC001 in breastfed term infants, in which mother-infant dyads received either lactation support or lactation support plus the probiotic for 21 days (21). We now present a secondary analysis of fecal samples collected from these infants. We hypothesized that given the capacity of *B. infantis* to outcompete other gut bacteria for HMOs, infants colonized with this strain in the first weeks of life would be stably colonized as long as human milk was provided. We also hypothesized that providing this strain would increase fecal short-chain fatty acids and decrease fecal pH, fecal HMO content, and fecal endotoxin concentrations.

## RESULTS

### Changes in the fecal microbiome.

**FIG 1:**
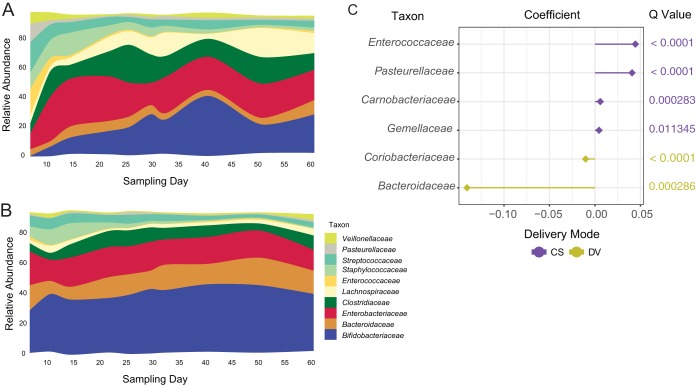
Mean relative abundance of predominant bacteria in CON infants delivered by CS (A) or DV (B). (C) Differentially abundant families were identified by using MaAsLin. Points are colored with respect to the delivery mode associated with their being differentially abundant: yellow, DV; purple, CS.

Among the infants whose mothers received lactation support alone (no probiotic for the infant [CON]), 22 out of 32 achieved measurable levels of *Bifidobacterium* colonization (>1% *Bifidobacterium*) in the first 60 days of life; however, only 10 infants maintained populations of *Bifidobacterium* greater than 50% of the total community, despite exclusive breastfeeding during this time period. Representative *Bifidobacterium* isolates from infants were predominantly *B. longum* subsp. *longum* and *B. breve* (see Table S1 in the supplemental material). When multiple confounding factors (subject, sampling day, mode of delivery) were corrected by multivariate linear modeling using MaAsLin, caesarean section (CS) delivery significantly altered the composition of the breastfed infant fecal microbiome over the first 60 days of life (Fig. 1A). Infants delivered vaginally (DV) had a higher abundance of *Bacteroidaceae* and lower abundances of *Enterococcaceae*, *Pasteurellaceae*, *Carnobacteriaceae*, and *Gemellaceae* compared to CS infants (Fig. 1B and C; Table 1).

10.1128/mSphere.00501-17.3 TABLE S1  *Bifidobacterium* isolates from feces of control infants. Download TABLE S1, PDF file, 0.03 MB. Copyright © 2017 Frese et al. 2017 Frese et al. This content is distributed under the terms of the Creative Commons Attribution 4.0 International license.

**TABLE 1:** Relative abundances of the most abundant taxonomic groups (at the family level) in infants, by birth mode and study group

Postnatal day	Organism	% mean relative abundance (SD) in study group^a^			
		CS-CON	DV-CON	CS-EVC001-fed	DV-EVC001-fed
6	*Bifidobacteriaceae*	0.09 (0.05)	28.99 (31.92)	19 (36.37)	17.11 (25.05)
6	*Bacteroidaceae*	4.88 (14.63)	15.87 (21.19)	0 (0)	16.25 (20.94)
6	*Enterobacteriaceae*	9.45 (14.96)	24.01 (23.07)	13.18 (28.17)	30 (29.1)
6	*Clostridiaceae*	1.3 (2.7)	2.87 (9.27)	4.4 (8.94)	4.13 (14.55)
6	*Enterococcaceae*	19.96 (20.84)	2.78 (8.59)	21.45 (29.35)	3.94 (6.6)
6	*Lactobacillaceae*	0.03 (0.07)	0.06 (0.17)	1.51 (4.48)	0.06 (0.22)
6	*Staphylococcaceae*	9.23 (10.54)	6.22 (7.28)	5.91 (8.39)	5.03 (5.9)
6	*Streptococcaceae*	26.44 (23.3)	8.35 (13.9)	15.58 (27.82)	10.29 (14.54)
6	*Veillonellaceae*	9.12 (13.01)	1.33 (2.85)	0.33 (0.78)	0.7 (1.76)
6	Other bacteria	19.52 (18.49)^b^	9.51 (11.71)	18.64 (22.13)	12.48 (16.42)

10	*Bifidobacteriaceae*	6.91 (18.61)	36.88 (30.73)	79.62 (30.3)	84.95 (9.49)
10	*Bacteroidaceae*	4.02 (12.03)	8.21 (9.89)	0.01 (0.01)	0.14 (0.48)
10	*Enterobacteriaceae*	30.94 (22.23)	16.19 (20.69)	3.96 (6.45)	2.15 (3.4)
10	*Clostridiaceae*	13.31 (17)	3.8 (8.49)	0.13 (0.27)	0 (0.01)
10	*Enterococcaceae*	6.61 (9.78)	0.56 (1.8)	4.09 (11.1)	0.01 (0.02)
10	*Lactobacillaceae*	0.06 (0.17)	1.62 (6.26)	0.03 (0.08)	0.03 (0.11)
10	*Staphylococcaceae*	12.08 (6.21)	8.87 (9.41)	3.84 (4.31)	6.24 (4.91)
10	*Streptococcaceae*	11.43 (17.4)	7.87 (11.93)	7.12 (13.77)	4.89 (3.3)
10	*Veillonellaceae*	6.26 (11.69)	3.32 (10.52)	0.6 (1.18)	0.48 (1.62)
10	Other bacteria	8.37 (10.37)	12.68 (22.96)	0.62 (0.49)	1.09 (1.78)

14	*Bifidobacteriaceae*	12.24 (24.12)	36.46 (33.27)	78.56 (31.59)	86.37 (8.36)
14	*Bacteroidaceae*	7.22 (15.21)	6.65 (9.38)	0.01 (0.01)	1.08 (3.08)
14	*Enterobacteriaceae*	33.33 (26.42)	21.1 (22.6)	5.78 (14.54)	1.51 (2.66)
14	*Clostridiaceae*	8.77 (11.29)	8.03 (11.88)	0.12 (0.22)	0 (0.01)
14	*Enterococcaceae*	1.38 (1.54)	0.43 (1.02)	6.08 (16.55)	0.02 (0.04)
14	*Lactobacillaceae*	4.14 (11.72)	1.43 (5.78)	0.02 (0.07)	0.01 (0.02)
14	*Staphylococcaceae*	14.1 (12.08)	9.82 (9.72)	4.99 (3.63)	5.86 (4.34)
14	*Streptococcaceae*	7.58 (9.58)	7.18 (12.29)	2.62 (2)	3.55 (2.57)
14	*Veillonellaceae*	3.28 (6.64)	1.28 (2.36)	1.2 (3.05)	0.87 (2.68)
14	Other bacteria	7.96 (16.71)	7.61 (12.65)	0.63 (0.65)	0.73 (0.91)

21	*Bifidobacteriaceae*	17.4 (24.6)	38.74 (35.47)	89.86 (5.12)	86.16 (14.71)
21	*Bacteroidaceae*	6.91 (13.49)	10.44 (14.59)	0.01 (0.01)	2.2 (6.41)
21	*Enterobacteriaceae*	31.02 (22.31)	21.32 (22.13)	0.91 (1.14)	0.98 (1.47)
21	*Clostridiaceae*	14.4 (18.50)	8.87 (12.21)	0.11 (0.27)	0 (0.01)
21	*Enterococcaceae*	1.22 (1.04)	0.38 (1.01)	1.98 (3.44)	0.05 (0.11)
21	*Lactobacillaceae*	0.38 (0.89)	0.65 (2.59)	0.02 (0.05)	0.08 (0.26)
21	*Staphylococcaceae*	5.91 (5.24)	3.51 (4.03)	2.85 (3.34)	1.83 (2.98)
21	*Streptococcaceae*	6.34 (11.92)	5.42 (6.53)	3.2 (2.63)	4.66 (6.2)
21	*Veillonellaceae*	1.22 (2.24)	1.14 (1.75)	0.42 (0.91)	0.9 (2.79)
21	Other bacteria	15.19 (21.57)	9.53 (12.20)	0.64 (0.54)	3.13 (9.81)

25	*Bifidobacteriaceae*	21.09 (28.05)	39.26 (36.05)	83.05 (26.31)	88.17 (10.97)
25	*Bacteroidaceae*	7.16 (14.12)	10.77 (14.55)	0 (0.01)	1.26 (2.51)
25	*Enterobacteriaceae*	23.34 (23.4)	20.79 (22.34)	3.97 (8.02)	2.72 (3.95)
25	*Clostridiaceae*	23.43 (28.53)	9.16 (13.9)	0.27 (0.68)	0 (0)
25	*Enterococcaceae*	0.89 (0.96)	0.33 (1.08)	4.67 (11.96)	0.17 (0.43)
25	*Lactobacillaceae*	1.31 (3.31)	0.42 (1.29)	0.02 (0.04)	0.25 (0.83)
25	*Staphylococcaceae*	4.67 (4.6)	2.38 (2.88)	1.64 (1.91)	1.02 (1.33)
25	*Streptococcaceae*	3.81 (2.68)	6.8 (11.54)	3.29 (3.22)	3.16 (3.78)
25	*Veillonellaceae*	1.15 (1.96)	1 (2.03)	1.88 (2.92)	1.08 (2.83)
25	Other bacteria	13.14 (18.78)	9.08 (9.51)	1.20 (1.76)	2.16 (5.76)

29	*Bifidobacteriaceae*	29.25 (33.47)	42.62 (38.43)	77.97 (29.18)	85.01 (13.16)
29	*Bacteroidaceae*	6.3 (12.11)	9.35 (13.26)	0.01 (0.01)	3.09 (6.35)
29	*Enterobacteriaceae*	15.9 (16.53)	21.28 (22.02)	4.84 (9.77)	2.39 (3.7)
29	*Clostridiaceae*	17.25 (22.33)	8.66 (13.13)	0.67 (1)	0.04 (0.16)
29	*Enterococcaceae*	0.67 (0.89)	0.3 (0.61)	5.97 (14.05)	0.3 (0.54)
29	*Lactobacillaceae*	0.96 (1.83)	0.58 (1.55)	0.4 (1.09)	1 (3.22)
29	*Staphylococcaceae*	5.15 (5.89)	1.48 (1.95)	0.94 (1.27)	0.45 (0.63)
29	*Streptococcaceae*	5.85 (6.67)	5.16 (11.69)	2.8 (3.27)	3.19 (3.5)
29	*Veillonellaceae*	1.77 (4.13)	0.95 (2.08)	3.25 (4.27)	1.53 (3.27)
29	Other bacteria	16.90 (23.17)	9.60 (10.42)	3.16 (3.09)	2.98 (5.12)

32	*Bifidobacteriaceae*	24.8 (32.37)	40.27 (34.56)	83.3 (15.6)	88.15 (10.08)
32	*Bacteroidaceae*	3.97 (11.21)	11.95 (14.9)	0 (0.01)	0.88 (1.99)
32	*Enterobacteriaceae*	24.63 (22.24)	19.99 (24.09)	2.26 (3.25)	2.88 (4.27)
32	*Clostridiaceae*	12.54 (16.95)	8.9 (15.47)	0.33 (0.71)	0.01 (0.03)
32	*Enterococcaceae*	0.51 (0.41)	0.21 (0.39)	3.47 (6.84)	0.69 (2.22)
32	*Lactobacillaceae*	2.76 (5.77)	0.41 (0.97)	0.98 (2.76)	1.34 (3.42)
32	*Staphylococcaceae*	3.55 (2.73)	1.52 (3)	0.56 (0.84)	0.42 (0.48)
32	*Streptococcaceae*	4.82 (1.38)	5.18 (8.22)	3.98 (4.55)	3.49 (3.98)
32	*Veillonellaceae*	1.05 (1.05)	1.31 (2.61)	2.99 (4.22)	0.77 (2.26)
32	Other bacteria	21.38 (26.33)	10.26 (10.56)	2.11 (2.26)	1.36 (1.82)

40	*Bifidobacteriaceae*	38.27 (38.61)	43.98 (37.26)	87.45 (6.55)	87.6 (11.63)
40	*Bacteroidaceae*	3.4 (9.9)	11.39 (14.32)	0.01 (0.01)	2.24 (4.7)
40	*Enterobacteriaceae*	22.2 (18.23)	18.96 (21.98)	2.33 (2)	3.44 (7.44)
40	*Clostridiaceae*	10.66 (20.36)	6.91 (11.35)	0.23 (0.49)	0.03 (0.1)
40	*Enterococcaceae*	0.34 (0.37)	0.65 (2.65)	2.16 (1.93)	0.34 (0.85)
40	*Lactobacillaceae*	1.66 (2.85)	0.83 (1.86)	0.49 (0.81)	0.27 (0.62)
40	*Staphylococcaceae*	2.65 (2.36)	0.65 (0.93)	0.29 (0.46)	0.41 (0.6)
40	*Streptococcaceae*	6.57 (11.72)	3.19 (4.11)	1.38 (1.87)	3.39 (2.94)
40	*Veillonellaceae*	3.63 (8.53)	1.49 (2.69)	1.07 (1.74)	0.69 (2.45)
40	Other bacteria	10.64 (17.05)	11.96 (15.59)	4.60 (4.85)	1.58 (2.14)

50	*Bifidobacteriaceae*	26.31 (33.96)	42.09 (33.34)	83.35 (9.02)	88.94 (9.54)
50	*Bacteroidaceae*	5.23 (9.84)	15.11 (15.56)	0.01 (0.01)	3.48 (6.89)
50	*Enterobacteriaceae*	25.8 (15.89)	19.56 (19.87)	2.58 (2.37)	2.03 (2.28)
50	*Clostridiaceae*	19.27 (21.38)	3.53 (7.54)	0.47 (0.72)	0.03 (0.08)
50	*Enterococcaceae*	0.42 (0.44)	0.77 (2.35)	2.35 (2.52)	0.35 (0.88)
50	*Lactobacillaceae*	1.55 (3.09)	1.36 (3.66)	0.69 (1.16)	0.56 (1.36)
50	*Staphylococcaceae*	1.7 (1.64)	0.46 (0.82)	0.26 (0.41)	0.29 (0.51)
50	*Streptococcaceae*	3.85 (3.32)	2.54 (3.24)	2.01 (2.3)	2.01 (1.93)
50	*Veillonellaceae*	1.99 (2.44)	1.11 (1.77)	2.93 (4.25)	0.75 (2.21)
50	Other bacteria	13.86 (16.35)	13.46 (18.09)	5.38 (4.24)	1.56 (1.56)

60	*Bifidobacteriaceae*	29.37 (28.77)	36.57 (32.48)	78.99 (15.51)	85.56 (11.88)
60	*Bacteroidaceae*	9.48 (18.79)	13.09 (15.75)	0 (0)	3.56 (6.42)
60	*Enterobacteriaceae*	19.37 (8.46)	14.69 (13.03)	3.48 (2.97)	3.53 (5.47)
60	*Clostridiaceae*	9.86 (10.92)	8.47 (15.12)	0.69 (1.01)	0.03 (0.07)
60	*Enterococcaceae*	1.1 (1.52)	0.19 (0.45)	3.27 (3.34)	0.4 (0.79)
60	*Lactobacillaceae*	2.1 (3.3)	0.91 (2.14)	0.53 (0.96)	0.33 (0.69)
60	*Staphylococcaceae*	1.32 (2.12)	0.56 (0.86)	0.34 (0.5)	0.36 (0.81)
60	*Streptococcaceae*	5.36 (7.82)	4.39 (5.83)	1.43 (1.21)	2.77 (3.27)
60	*Veillonellaceae*	2.21 (3.08)	5.22 (7.86)	3.97 (5.41)	0.3 (0.5)
60	Other bacteria	19.81 (23.49)	15.88 (15.18)	7.31 (7.91)	3.16 (4.94)

a Group abbreviations indicate the route of delivery (CS or DV) and the treatment group. CON, control.

b Values for the “Other bacteria” rows represent all other, less abundant, bacterial families.

**FIG 2:**
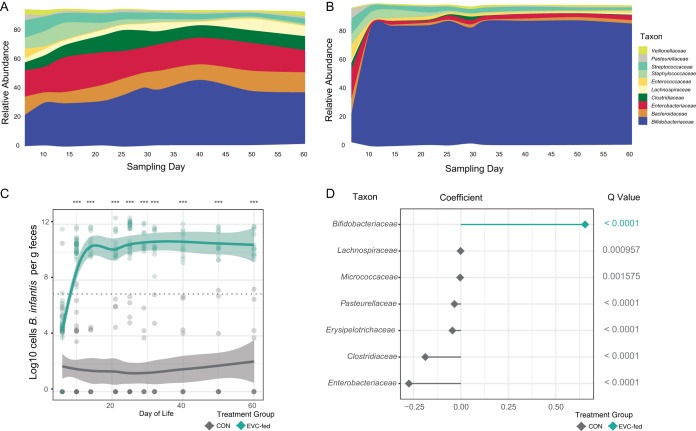
Mean relative abundance of predominant taxa in feces of CON (A) and EVC001-fed (B) infants. (C) Log_10_ of *B. infantis* cells per gram of feces during the supplementation period (day 8 to 29) and through 60 days postnatal, with a Loess fit curve with confidence intervals. (D) Differentially abundant bacterial families identified by MaAsLin. ***, *P* < 0.001.

Among the infants fed *B. infantis* EVC001, fecal *Bifidobacteriaceae* were significantly higher in these infants than in the CON infants (Fig. 2A and B; Table 1). In addition, a species-specific quantitative PCR (qPCR) measurement showed significantly higher mean levels of *B. infantis* in supplemented infants (10.81 log_10_ cells per g of feces [standard deviation, or SD, 11.13]). The number of cells (reported as the log number of cells per gram of feces) at day 10 postnatal in the EVC001 treatment group (Fig. 2C) was higher than in CON infants, whose mean levels of *B. infantis* fell below the limit of detection throughout the 21-day feeding period (days 7 to 28 postnatal) and through 60 days postnatal (Fig. 2C). After the baseline sample (*P* = 0.431, Holm-Sidak adjusted), the difference was significant between the two groups from day 10 through day 60 (*P* < 0.0001, Holm-Sidak adjusted). The relative abundances of *Enterobacteriaceae*, *Clostridiaceae*, *Erysipelotrichaceae*, *Pasteurellaceae*, *Micrococcaceae*, and *Lachnospiraceae* were markedly diminished in supplemented infants compared with CON infants based on a comparison using MaAsLin to account for subject, sampling day, and delivery mode as confounding variables (Fig. 2D). Mean relative abundances of the dominant taxonomic families (± SD) are shown in Table 1 for each day, each delivery mode, and each treatment group.

**FIG 3:**
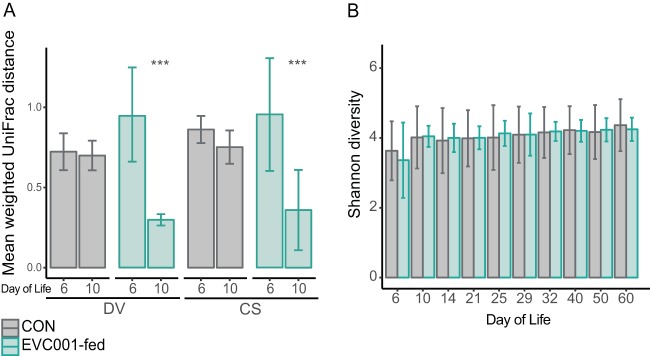
(A) Mean weighted UniFrac distance (± SD) between fecal samples from CON infants and from infants in the EVC001 supplementation group. (B) Mean Shannon diversity index (± SD). Note that *B. infantis* EVC001 was fed from day 7. ***, *P* < 0.001.

**FIG 4:**
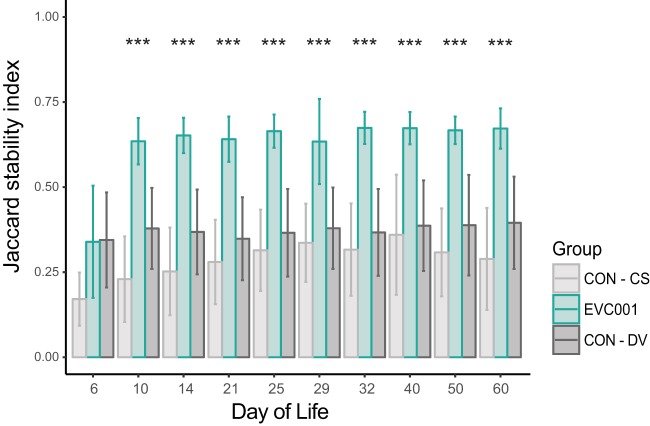
Jaccard stability index for EVC001-fed and CON infants; significant differences were calculated between EVC001 and other groups by using multiple *t* tests with the Holm-Sidak correction. ***, *P* < 0.001.

To examine the effect of feeding *B. infantis* EVC001 on the fecal microbiome of CS infants and whether any changes resolved the effect of CS on the infant fecal microbiome, the weighted UniFrac distances were compared between delivery modes (CS versus DV) in probiotic-supplemented infants. After supplementation, the mean weighted UniFrac distance among samples within delivery groups decreased significantly from that of baseline samples (*P* < 0.001, Holm-Sidak-corrected multiple-comparisons test) (Fig. 3A). Despite these compositional changes in the overall microbial community, the Shannon diversity index was not significantly different between EVC001-fed and CON infants (*P* > 0.05, Holm-Sidak-corrected multiple-comparisons test) (Fig. 3B). Community stability was compared over this time period using an abundance-weighted Jaccard index. The fecal microbiome of infants fed with *B. infantis* EVC001 was more stable over time than that of CON infants, and this higher stability persisted through postnatal day 60 (*P* < 0.001 throughout; Holm-Sidak-corrected multiple-comparisons test) (Fig. 4). Even though *B. infantis* EVC001 feeding stopped at postnatal day 28, infants fed *B. infantis* EVC001 maintained significantly higher abundances of fecal *B. infantis* for 30 days after supplementation compared to control infants, based on 16S rRNA sequencing and species-specific qPCR.

### Changes in fecal markers of dysbiosis.

**FIG 5:**
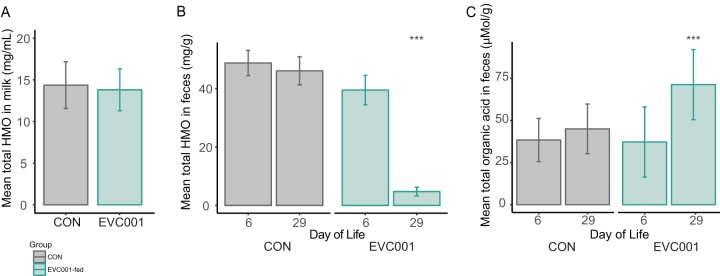
(A) Mean concentration of HMOs (in milligrams per milliliter, ± SD) in milk among mothers in the study. (B) Mean concentration of fecal HMOs (in milligrams per gram of feces, ± SD) in infant stools at day 6 and day 29. (C) Mean organic acids (lactate and SCFA) in fecal samples at day 6 or day 29 (in micromoles per gram, ± SD). Note that *B. infantis* EVC001 was fed from day 7. ***, *P* < 0.001.

To examine whether the HMO composition and concentration in mother’s milk may have influenced the fecal microbiome composition, milk samples from mothers in the study were analyzed by liquid chromatography-mass spectrometry. There were no differences in the concentrations of HMOs in milk between mothers in the two study groups (Fig. 5A) (*P* = 0.41, Mann-Whitney test) nor in the concentration of 2′-fucosyllactose, a key indicator of maternal mutations in the *FUT2* gene that may influence the infant fecal microbiome (4) (*P* = 0.83, Mann-Whitney test; *n* = 34 and 32, respectively) (Table 2). The composition and concentration of HMOs in infant feces are markers of HMO consumption by gut microbes and/or absorption (22). The mean fecal HMO concentration in samples from *B. infantis* EVC001-fed infants (4.75 mg/g [SD, 8.6]) was 10-fold lower than in samples from CON infants at day 29 postnatal (46.08 mg/g [SD, 26.78]; *P* < 0.001 by Tukey’s multiple-comparison test) (Fig. 5B), consistent with increased utilization of ingested HMOs by the probiotic *B. infantis* EVC001. Nearly all HMOs in the stools decreased after colonization with *B. infantis* EVC001 (Table 3).

**TABLE 2:** HMO profiles in milk collected at postnatal day 21

HMO^a^	Mean (SD) HMO concn (mg/ml)		Corrected *P* value^b^
	CON (*n* = 32)	EVC001-fed (*n* = 34)	
Total	14.37 (2.80)	13.81 (0.43)	NS
2′-FL	1.62 (0.97)	1.71 (1.04)	NS
3′-FL	0.5 (0.33)	0.55 (0.05)	NS
3000	0.18 (0.17)	0.18 (0.06)	NS
LDFT	0.11 (0.02)	0.15 (0.03)	NS
LNT, LNnT	3.35 (0.31)	2.89 (0.24)	NS
LNFP I	2.05 (0.31)	1.85 (0.27)	NS
LNFP II, III, V	1.12 (0.13)	1.09 (0.12)	NS
3′-SLN, 6′-SLN	0.01 (0)	0.01 (0)	NS
LNH, LNnH	0.47 (0.05)	0.4 (0.03)	NS
LNDFH I, II	0.21 (0.03)	0.23 (0.02)	NS
3′-SL, 6′-SL	1.54 (0.07)	1.62 (0.07)	NS
MFLNH, IFLNH	0.78 (0.04)	0.77 (0.04)	NS
4300	0.05 (0)	0.04 (0)	NS
4220	0.31 (0.02)	0.31 (0.01)	NS
5310	0.18 (0.01)	0.15 (0.01)	NS
LSTa, b, c	0.31 (0.02)	0.31 (0.02)	NS
4230	0.08 (0.01)	0.09 (0.01)	NS
5320	0.2 (0.01)	0.18 (0.01)	NS
6410	0.14 (0.01)	0.14 (0.01)	NS
3111	0.01 (0)	0.01 (0)	NS
5330	0.12 (0.01)	0.11 (0)	NS
4201	0.32 (0.03)	0.3 (0.03)	NS
6420	0.11 (0.01)	0.1 (0.01)	NS
5340	0.02 (0)	0.02 (0)	NS
4211	0.47 (0.03)	0.47 (0.02)	NS
6440	0.02 (0)	0.02 (0)	NS
5301	0.01 (0)	0.01 (0)	NS
5311	0.04 (0)	0.04 (0)	NS
6401	0.02 (0)	0.02 (0)	NS
5321	0.02 (0)	0.02 (0)	NS
6430	0.02 (0)	0.02 (0)	NS
4221	0.01 (0)	0.01 (0)	NS
5331	0 (0)	0 (0)	NS

a Abbreviations: 2′-FL, 2′-fucosyllactose; 3′-FL, 3′-fucosyllactose; LDFT, lacto-difucosyltetraose; LNT, lacto-*N*-tetraose; LNnT, lacto-*N*-neotetraose; LNFP, lacto-*N*-fucosyllpentose; SLN, sialyllactosamine; LNH, lacto-*N*-hexose; LNnH, lacto-*N*-neohexose; DFLNH, difucosyllacto-*N*-hexose; MFLNH, mono-fucosyllacto-*N*-hexose; IFLNH, isomeric fucoysllactosyl-*N*-hexose; LST, sialyl-lacto*-N*-tetraose; LSTa, b, c, isomers of LST. Structural formula for unnamed structures is hexose | hexose-*N*-acetylglucosamine/galactosamine | fucose | sialic acid.

b *P* values were determined with a multiple *t* test and the Holm-Sidak correction. NS, not significant.

**TABLE 3:** Infant fecal HMO profiles at baseline (postnatal day 6) and postnatal day 29

HMO^a^	Mean (SD) HMO concn (mg/ml)				Adjusted *P* value^b^
	CON on:		EVC001-fed on:		
	Day 6 (n = 32)	Day 29 (n = 32)	Day 6 (n = 34)	Day 29 (n = 34)	
1101	0.16 (0.25)	0.08 (0.13)	0.1 (0.09)	0 (0.01)	0.0664
2001	6.37 (4.45)	5.12 (5.34)	5.07 (4.55)	0.39 (1.91)	0.0056
2020	0.45 (0.43)	0.6 (0.59)	0.69 (0.84)	0.08 (0.23)	0.0688
2-FL	1.97 (1.93)	2.01 (2)	1.57 (1.94)	0.03 (0.14)	0.0034
3000	0.68 (1.36)	0.53 (1.55)	1.3 (2.34)	0.01 (0.02)	0.4457
3100	7.88 (6.79)	7.56 (11.15)	7.35 (8.15)	0.47 (1.54)	0.0664
3101	4.25 (2.81)	2.16 (1.67)	3.11 (2.35)	0.1 (0.43)	0.0034
LNFP I	9.62 (7.41)	6.71 (7.08)	5.97 (6.43)	0.24 (1.27)	0.0056
LNFP III, IV, V	4.97 (2.72)	5.71 (3.73)	3.75 (3.13)	0.12 (0.4)	<0.0001
3111	0.05 (0.05)	0.03 (0.04)	0.06 (0.07)	0.00 (0)	0.2198
3120	0.84 (0.66)	1.15 (1.08)	0.77 (0.66)	0.04 (0.14)	0.0001
3′-FL	0.99 (0.88)	2.27 (2.06)	1.23 (1.28)	0.05 (0.18)	0.0001
4200	0.7 (0.79)	1.23 (1.92)	0.76 (1.36)	0.39 (0.45)	0.2198
4201	1.24 (1.29)	0.96 (1.4)	0.89 (0.85)	0.09 (0.34)	0.0607
4210	1.96 (1.35)	2.61 (2.46)	1.66 (1.82)	0.3 (0.55)	0.0015
4211	1.34 (0.95)	1.55 (1.6)	1.13 (1.01)	0.13 (0.55)	0.0015
4220	1.01 (0.56)	1.53 (1.01)	0.79 (0.6)	0.11 (0.23)	<0.0001
4221	0.1 (0.09)	0.07 (0.11)	0.06 (0.06)	0.01 (0.02)	0.046
4230	0.21 (0.21)	0.4 (0.45)	0.18 (0.21)	0.02 (0.05)	0.0014
4300	0.28 (0.2)	0.25 (0.28)	0.18 (0.29)	0.82 (0.99)	<0.0001
5301	0.14 (0.12)	0.08 (0.13)	0.09 (0.08)	0.02 (0.03)	0.2198
5310	0.94 (0.58)	0.92 (0.95)	0.76 (0.87)	0.4 (0.44)	0.1916
5311	0.26 (0.19)	0.12 (0.12)	0.18 (0.18)	0.01 (0.03)	0.0664
5320	0.97 (0.61)	0.95 (0.89)	0.69 (0.65)	0.26 (0.25)	0.0117
5321	0.1 (0.08)	0.07 (0.06)	0.07 (0.07)	0 (0.01)	0.0047
5330	0.28 (0.31)	0.37 (0.39)	0.29 (0.32)	0.13 (0.17)	0.0838
5331	0.02 (0.02)	0.01 (0.01)	0.01 (0.01)	0 (0)	0.2198
5340	0.06 (0.05)	0.06 (0.06)	0.05 (0.06)	0.02 (0.03)	0.1282
6401	0.05 (0.06)	0.03 (0.05)	0.04 (0.06)	0.01 (0.01)	0.2674
6410	0.41 (0.34)	0.47 (0.61)	0.36 (0.51)	0.31 (0.36)	0.4457
6420	0.28 (0.25)	0.36 (0.39)	0.22 (0.25)	0.18 (0.18)	0.2198
6430	0.1 (0.09)	0.06 (0.07)	0.06 (0.07)	0.01 (0.03)	0.0896
6440	0.06 (0.05)	0.06 (0.05)	0.04 (0.04)	0.01 (0.02)	0.0023
Total	48.74 (23.79)	46.08 (26.78)	39.48 (28.42)	4.75 (8.6)	<0.0001

a 2′-FL, 2′-fucosyllactose; 3′-FL, 3′-fucosyllactose; LNFP, lacto-*N*-fucosyllpentose. The structural formula for unnamed structures is hexose | hexose-*N*-acetylglucosamine/galactosamine | fucose | sialic acid.

b Day 29 results were compared with a multiple *t* test with the Holm-Sidak correction.

Liquid chromatography-mass spectrometry was also used to measure lactate and short-chain fatty acids (SCFAs) in feces, as these are the primary metabolic end products of *Bifidobacterium* HMO fermentation. Infants fed *B. infantis* EVC001 had significantly increased fecal lactate and acetate (Fig. 5C; Table 4). Infants fed *B. infantis* EVC001 had significantly greater total fecal organic acids than CON infants at day 29 (71.41 ± 20.75 μmol/g versus 45.00 ± 14.73 μmol/g; adjusted *P*  < 0.001, multiple *t* test with Holm-Sidak correction).

**TABLE 4:** Infant fecal lactate and short-chain fatty acid profiles at baseline (postnatal day 6) and postnatal day 29

Molecule	Mean (SD) amt in feces (μmol/g of feces)				Adjusted *P* value^a^
	CON on:		EVC001-fed on:		
	Day 6 (*n* = 32)	Day 29 (*n* = 32)	Day 6 (*n* = 34)	Day 29 (*n* = 34)	
Lactate	4.39 (5.4)	7.45 (9.15)	6.59 (11.2)	26.04 (14.86)	<0.001
Acetate	21.12 (8.68)	25.06 (9.38)	19.63 (11.44)	36.29 (11.47)	<0.001
Butyrate	0.15 (0.51)	0.51 (0.74)	0.46 (1.33)	0.1 (0.21)	0.014
Formate	11.68 (3.67)	11.24 (3.65)	8.74 (3.92)	7.25 (3.72)	<0.001
Hexanoate	0.02 (0.04)	0.01 (0)	0.01 (0)	0.01 (0.01)	0.674
Isobutyrate	0.06 (0.2)	0.07 (0.23)	0.06 (0.14)	0.05 (0.11)	0.798
Isovalerate	0.1 (0.17)	0.06 (0.02)	0.1 (0.1)	0.07 (0.09)	0.798
Propionate	0.82 (1.17)	0.55 (0.67)	1.57 (2.32)	1.4 (2.45)	0.292
Valerate	0.05 (0.2)	0.01 (0.01)	0.03 (0.07)	0.04 (0.18)	0.798
Total	38.41 (12.79)	45 (14.73)	37.22 (20.84)	71.41 (20.75)	<0.001

a Comparisons between samples collected at day 29 were made via multiple *t* tests with the Holm-Sidak correction.

**FIG 6:**
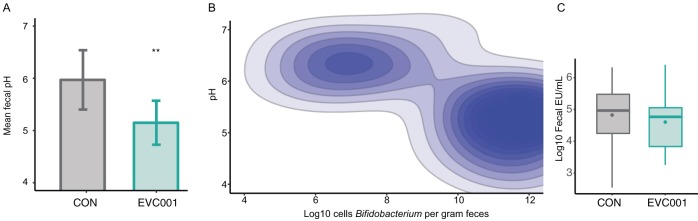
Biochemistry changes associated with EVC001 colonization. (A) Mean fecal pH (± SD) from infants fed EVC001 and CON infants. (B) Fecal *Bifidobacterium* counts (log_10_ cells per gram of feces) correlate with pH. (C) Median log_10_ fecal endotoxin (EU per milliliter) from supplemented (EVC001) and UNS infants. Points represent mean values. **, *P* < 0.01.

To test whether fecal pH changed in tandem with increased lactate and acetate concentrations, the pH of the fecal samples at postnatal day 21 from 18 randomly chosen CON infants was measured. The mean fecal pH of these samples was 5.97 ± 0.57. In contrast, feces from 18 randomly sampled *B. infantis* EVC001-fed infants at day 21 had a mean pH of 5.15 ± 0.42, which was significantly lower than the pH of feces from CON infants (*P* < 0.01, Mann-Whitney test) (Fig. 6A). Comparison between infant groups showed absolute *Bifidobacterium* populations in infant stools were negatively correlated with fecal pH (Spearman’s ρ = −0.62, *P* < 0.01) (Fig. 6B). Comparing weighted UniFrac distance matrices, the pH was also found to be a significant discriminator of community composition (Mantel test, *R* = 0.32, *P* = 0.002). *Actinobacteria* (the phylum containing bifidobacteria) were significantly and negatively correlated with pH (Spearman’s ρ = −0.54, *P* = 0.018, Bonferroni corrected), while *Proteobacteria* were significantly and positively correlated with pH (Spearman’s ρ = 0.65, *P* < 0.001, Bonferroni corrected).

Endotoxin, an important outer membrane component of Gram-negative organisms (e.g., *Proteobacteria* and *Bacteroidetes*), was significantly and positively correlated with the relative abundance of *Enterobacteriaceae* (Spearman’s ρ = 0.51, *P* < 0.001) and was negatively correlated with *Bifidobacteriaceae* abundance (Spearman’s ρ = −0.42, *P* < 0.001). Individual infants had high interindividual variation, and the mean log_10_ fecal endotoxin units (EU) per milliliter was not significantly lower in *B. infantis* EVC001-fed infants (4.58 ± 0.89 log_10_ EU/ml) than in CON infants (4.90 ± 0.91 log_10_ EU/ml; *P* = 0.92, Mann-Whitney test) (Fig. 6C). However, when a robust nonlinear regression was used to detect and remove outliers (ROUT [23]), the difference was significant (4.25 ± 0.61 log_10_ EU/ml in *B. infantis* EVC001-fed infants and 4.58 ± 0.75 log_10_ EU/ml in CON infants; *P* = 0.04). A stronger effect was found when *Bifidobacteriaceae* relative abundance was taken into account in all infants tested. Infants with high populations of *Bifidobacteriaceae* (>50%; *n* = 46) had significantly lower endotoxin concentrations than infants with low *Bifidobacteriaceae* abundance levels (<50%; *n* = 13), with 4.68 log_10_ EU/ml versus 5.36 log_10_ EU/ml (*P* = 0.007, Mann-Whitney test).

## DISCUSSION

Neonates are rapidly colonized by the organisms they encounter at parturition, through exposure to vaginal and fecal microbes during vaginal delivery or by exposure to skin and hospital-associated surfaces during caesarean section delivery (24, 25). Despite exclusive breastfeeding, in this study many CON infants remained uncolonized by *Bifidobacterium* throughout the initial 60-day study period, and those that were colonized by this genus harbored species that were distinct from *B. infantis*, the predominant species found in many breastfed infants in resource-poor countries (15, 26).

The most striking finding of this study was the stable colonization of *B. infantis* well beyond the supplementation period. Even though *B. infantis* EVC001 was not provided after day 28, fecal bifidobacteria, which we confirmed as *B. infantis* EVC001 based on quantitative PCR, remained the dominant organism in these infants at day 60 (Fig. 2). We postulate that this prolonged colonization, which has been rarely observed in probiotic studies (18), is a function of the ancient adaptations of *B. longum* subsp. *infantis* to HMOs (27), and some of these adaptations provide a specific ecological advantage over those of other gut microbes (e.g., *Bacteroides*) (28). Thus, both probiotic inoculation and continued provision of a selective nutrient (HMOs) for the probiotic strain are likely to be essential to long-term colonization; these findings are analogous to recent findings for *B. longum* subsp. *longum* in adults (18).

In the distal gut, carbohydrates play a major role in shaping the microbial community structure. HMOs are energetically expensive for the mother to produce and compose the third largest fraction of solids in human milk (19). Given that HMOs are not digested by the infant, they are abundant in the stool in the absence of *B. infantis* or other HMO-consuming microbes. This could represent a potential marker of intestinal dysbiosis (i.e., when the intestinal microbiome is dominated by non-HMO-consuming microbes, such as *Proteobacteria* and *Firmicutes*, the fecal HMO content is high) (22, 29, 30). A number of functions of HMOs have been proposed, from immune signaling to serving as prebiotics (31), and changes in the concentrations of HMOs in milk and microbiome compositions are associated with differential impacts on health outcomes (3). Here, the provision of *B. infantis* EVC001 markedly decreased fecal HMOs, consistent with high levels of consumption by *B. infantis* EVC001. The associated increases in lactate and acetate and decline in fecal pH were not surprising, given that the central metabolic pathway for *Bifidobacterium* yields lactate and acetate as primary products of fermentation (32).

In the absence of *Bifidobacterium*, the community that dominates the gut microbiome of infants in resource-rich countries is typically characterized as having low community stability and diminished colonization resistance, which are among the key ecosystem functions for the gut microbiome (33). In infants colonized by *B. infantis* EVC001, community stability is high and saturated, even at a low level of community diversity, compared with the adult gut ecosystem. Thus, we hypothesize that the evolutionary and ecological adaptations that have shaped the mother-infant-*B. infantis* relationship have converged on an optimum that maximizes community stability and the fermentative output of organic acids for the benefit of the host. Here, we found that a low fecal pH was negatively associated with *Proteobacteria*, whose presence in a gut community is considered a signature of dysbiosis (34). By producing lactate and acetate, *B. infantis* EVC001 is also able to convert indigestible carbohydrates—otherwise lost in the stool—into substrates that are actively transported by the host and play central roles in host overall energy balance, immune development, and support of colonic epithelial cell growth during a critical window of infant development (35, 36). Indeed, recent studies have shown that infants colonized with *B. infantis* at levels comparable to those of the infants supplemented with *B. infantis* EVC001 have improved health outcomes compared to infants with dysbiotic gut microbiomes (1) and that high levels of *B. infantis* are typical for healthy infants (37). Future studies will be necessary to elucidate the durability of this effect through later childhood and whether these effects have an impact on overall health later in life.

## MATERIALS AND METHODS

### Study subjects and design.

The details of the clinical trial design were reported previously (21). Briefly, mother-infant dyads were recruited in the Davis and Sacramento metropolitan region of Northern California (USA) (Table S2). Mothers received either lactation support or lactation support and 1.8 × 10^10^ CFU of activated *B. infantis* EVC001 (ATCC SD-7035; manufactured by Evolve Biosystems, Inc.) to feed their infants daily from postnatal day 7 to day 28. This strain of *B. infantis* was selected for its capacity to digest the full breadth of HMOs (38–40). *B. infantis* EVC001 was delivered as 156 mg of live bacteria (>1.8 × 10^10^ CFU) diluted in 469 mg of lactose as an excipient, and it was packaged in single-use sachets. Mothers were trained by lactation consultants to mix the contents of the sachet in 5 ml of expressed breast milk and feed this to the infant. The probiotic was stored at −20°C by the mothers during the study, and stability at −20°C was confirmed by plate counts. Subject sampling is illustrated in Fig. S1. The study and methods were approved by the UC Davis Institutional Review Board, and the study was registered at Clinicaltrials.gov (NCT02457338).

10.1128/mSphere.00501-17.1 FIG S1  Subject demographics, delineating whether mothers received antibiotics during labor (the subject ID is underlined) or whether infants received antibiotics at or prior to sampling time (triangles), and whether a mother received lactation support (gray) or lactation support and *B. infantis* EVC001 (teal). Download FIG S1, PDF file, 0.2 MB. Copyright © 2017 Frese et al. 2017 Frese et al. This content is distributed under the terms of the Creative Commons Attribution 4.0 International license.

10.1128/mSphere.00501-17.4 TABLE S2  Study population demographics. Download TABLE S2, PDF file, 0.2 MB. Copyright © 2017 Frese et al. 2017 Frese et al. This content is distributed under the terms of the Creative Commons Attribution 4.0 International license.

### Sample collection.

Fecal samples were collected at home on postnatal days 6 (baseline), 10, 14, 21, 25, 29, 32, 40, 50, and 60 as previously described (21). Infants from the EVC001-fed group (*n* = 34) and the control group (*n* = 32, CON) who maintained exclusive breastfeeding or abstained from routine use of nonstudy probiotics were included in this secondary analysis. Adequate sample volumes for the analysis were available for 66 infants (Fig. S1) who met these final study criteria. Stool samples were stored at −20°C in a home freezer and transferred on dry ice to a −80°C freezer for storage prior to DNA extraction. All individuals processing and analyzing samples were blinded to study group assignments.

### Molecular methods and analyses.

Total DNA was extracted from approximately 100 mg of feces by using the Zymo fecal DNA miniprep kit according to the manufacturer’s instructions (Zymo Research). Negative controls for detection of kit contamination were included and failed to produce visible PCR bands in an agarose gel but were analyzed as quality controls. Quantitative PCR was carried out using standard curves of known cultures prepared by serial dilution and extracted in the same manner as the fecal samples. Individual samples were analyzed in duplicate under the conditions listed in Table 5. Reactions were carried out in 20-µl volumes with PerfeCTa SYBR green FastMix ROX (QuantaBio; Beverly, MA USA) or TaqMan universal master mix II, with uracil-*N*-glycosylase (Thermo Fisher Scientific; Waltham, MA, USA), in 5 µl of extracted DNA in a QuantStudio 3 qPCR machine (Thermo Fisher Scientific; Waltham, MA, USA).

**TABLE 5:** Primers, probes, and cycling conditions used in this study

Primer name	Gene target	Taxon	Sequence (5′–3′)^a^	Concn (final, nM)	PCR conditions^b^	Reference(s)
Bif F	16S rRNA	*Bifidobacterium*	GCGTGCTTAACACATGCAAGTC	600	A	52
Bif R	16S rRNA	*Bifidobacterium*	CACCCGTTTCCAGGAGCTATT	600	A	52
Bif P	16S rRNA	*Bifidobacterium*	6-FAM–TCACGCATTACTCACCCGTTCGCC–BHQ1	250	A	52
BiLONF	16S rRNA	*B. longum* group	CAGTTGATCGCATGGTCTT	900	B	53
BiLONR	16S rRNA	*B. longum* group	TACCCGTCGAAGCCAC	900	B	53
BiLONSpP	16S rRNA	*B. longum* group	6-FAM–TGGGATGGGGTCGCGTCCTATCAG–TAMRA	250	B	53
Blon0915F	Blon0915	*B. longum* subsp. *infantis*	CGTATTGGCTTTGTACGCATTT	900	C	This study
Blon0915R	Blon0915	*B. longum* subsp. *infantis*	ATCGTGCCGGTGAGATTTAC	900	C	This study
BI915 PRB	Blon0915	*B. longum* subsp. *infantis*	6-FAM–CCAGTATGG–ZEN–CTGGTAAAGTTCACTGCA– 3IABkFQ	250	C	This study
515F	16S rRNA	Bacteria, universal	GTGYCAGCMGCCGCGGTAA	200	A	43, 44
806R	16S rRNA	Bacteria, universal	GGACTACNVGGGTWTCTAAT	200	A	43, 44
Probio-bifUni	ITS^c^ region	*Bifidobacterium*	CTKTTGGGYYCCCKGRYYG	1,000	A	54
Probio-bifRev	ITS region	*Bifidobacterium*	CGCGTCCACTMTCCAGTTCTC	1,000	A	54

a 6-FAM, 6-carboxyfluorescein; BHQ1, Black hole quencher 1; TAMRA, 6-carboxytetramethylrhodamine; ZEN, ZEN quencher; 3IABkFQ, Iowa Black fluorophore quencher.

b Reaction conditions: A, as described in the text; B, 2 min at 50°C, then 40 cycles of 15 s at 95°C and 60 s at 58°C; C, 2 min at 50°C, 10 min at 95°C, and then 40 cycles of 1 s at 95°C and 60 s at 60°C.

c ITS, internal transcribed spacer.

### Development of Blon_0915 primers.

A bidirectional BLAST search was used to compare type strains of *B. infantis* and *B. longum* to identify subspecies-specific genes. Candidate genes were screened against *B. infantis* EVC001 ATCC SD7035, and Blon_0915 was found to be present in both the type strain of *B. infantis* and EVC001 ATCC SD7035 and other closely related strains of *B. infantis* but not among other *Bifidobacterium* species. BLASTN searches confirmed these findings, indicating that Blon_0915 had little sequence homology among other *Bifidobacterium* sequences in the NCBI database. Primer3 (41, 42) was used to identify primer pairs with high efficiency and specificity for *B. infantis*. In comparison to the *B. longum* group primers used here (Table 5), primers Blon_0915F and Blon_0915R, when coupled with Blon_0915P, did not produce false amplification from infants who were not previously fed *B. infantis*. This was true even when tested in fecal samples from infants natively colonized by *B. longum*, which was independently verified by using genus-specific *Bifidobacterium* and *B. longum* group-specific qPCR primer sets (Fig. S2). The TaqMan reaction was carried out using the manufacturer’s instructions (Thermo, Fisher Scientific; Waltham, MA, USA), which included a preincubation step for 2 min at 50°C and then 10 min at 95°C, followed by 40 cycles of a two-step PCR for 15 s at 95°C and 60 s at 60°C for Blon_0915 primers; other primer/probe chemistries are outlined in Table 5.

10.1128/mSphere.00501-17.2 FIG S2  Cross-method validation of Blon_0915 qPCR methodology with infant fecal samples. DNA extracted from samples from infants in either the control group (subject 7071) or in the EVC001-fed group (subject 7007) was used to demonstrate that Blon_0915 primers fail to amplify other *B. longum* group species (e.g., *B. longum* subsp. *longum*, as shown here). DNA extracted from an infant fed EVC001 was also used to demonstrate that Blon_0915 primers amplify populations of *B. infantis* EVC001 only when fed *B. infantis* EVC001 and in populations, reflected by quantification using previously published *Bifidobacterium* genus-specific primers. Download FIG S2, PDF file, 0.2 MB. Copyright © 2017 Frese et al. 2017 Frese et al. This content is distributed under the terms of the Creative Commons Attribution 4.0 International license.

### Culture and identification of *Bifidobacterium* isolates.

Fecal *Bifidobacterium* isolates were obtained by serial dilution of feces in sterile phosphate-buffered saline (pH 7.0) and spread by plating on *Bifidobacterium* selective isolation medium (4). Plates were incubated anoxically at 37°C for 48 h, and 10 colonies were randomly selected and isolated per sample. The resulting strains were identified by PCR amplification, and the internal transcribed spacer amplicons were identified using primers and reaction conditions (Table 5) and then purified for Sanger sequencing at the UC Davis DNA Sequencing Core Facility.

### 16S rRNA sequencing and analysis.

The V4 region of the 16S rRNA gene was amplified and sequenced using primers 515f and 806r as previously described with recent modifications (43, 44) and as listed in Table 5, in a HEPA-filtered laminar flow cabinet dedicated to PCR preparation. Paired-end DNA (300 bp) sequencing was performed at the UC Davis Genome Center on an Illumina MiSeq system. Sequences were analyzed using QIIME 1.9.1 (45). Open-reference operational taxonomical unit (OTU) picking was performed using UCLUST at 97% identity (46). OTUs with a relative abundance of less than 0.005% were removed (47). Samples with fewer than 2,779 reads were omitted from analysis, which removed 10 samples and the negative-control samples (PCR and extraction control samples). After quality filtering, a mean of 9,216 (±4,505 [SD]) and a median of 7,749 reads were obtained per sample. The observed species index, Faith’s phylogenetic diversity index (48), and Shannon diversity index were used as metrics to compute alpha diversity. Weighted UniFrac distances were used as a beta diversity metric, in addition to the abundance-weighted Jaccard index, to calculate community compositional stability, congruent with previously described metrics of community stability (26, 49). Multivariate linear modeling (MaAsLin) was used to compare groups of samples at the family and genus level, correcting for subject, sampling day, treatment group, and delivery mode (50). In particular, taxonomic profiles at the family level of samples from day 10 to day 60 were imputed in MaAsLin, and the test was run to correct for subject, collection day, delivery mode (DV or CS), and treatment (EVC001-fed or CON). MaAsLin was run with a false-discovery rate of 0.05, a minimum of 0.0001 for feature relative abundance filtering, and a minimum of 0.01 for feature prevalence filtering.

### Biochemical measurements.

Breast milk collected on postnatal day 21 and fecal samples collected from baseline (day 6) to the end of EVC001 feeding (day 29) were analyzed for HMO composition as previously described (22) and also analyzed for fecal lactate and SCFA as previously described (51). To measure fecal pH, feces were diluted 1:10 in sterile water, mixed with a vortex mixer for 4 min, and centrifuged to precipitate solids (2 min, 12,000 relative centrifugal force). The supernatant was collected and its pH was measured (Oakton pH 700). Fecal endotoxin concentrations were measured by serial dilution in sterile, endotoxin-free water to within the reference range by using a Pierce LAL chromogenic endotoxin quantification kit (Thermo Fisher Scientific, Waltham, MA). Samples were measured in duplicate.

### Statistical methods.

Statistical comparisons were conducted as described in the figure legends or in the text. Linear modeling, analysis of variance, Mann-Whitney tests, and multiple comparisons (with Holm-Sidak correction) were performed using GraphPad Prism 7.0 (GraphPad Software, Inc. La Jolla, CA) or R (version 3.2.3).

### Accession number(s).

Sequencing libraries generated in this study have been deposited with the NCBI SRA (PRJNA390646) and are publicly available.
