# Supplementary material for: Persistence of Supplemented Bifidobacterium longum subsp. infantis EVC001 in Breastfed Infants
Source: mSphere. 2017 Dec 6;2(6):e00501-17. doi: 10.1128/mSphere.00501-17 (PMC5717325; doi:10.1128/mSphere.00501-17)
Supplement: TABLE S1 [file sph006172417st3.pdf]

TABLE S1

**Species**	**Proportion of isolates[a]**
B. longum* subsp. *longum	57.28%
B. breve	27.18%
B. animalis* subsp. *lactis	7.28%
B. bifidum	6.80%
B. dentium	0.97%
B. tsurumiense (low confidence)	0.49%

[a]There were 206 isolates of *Bifidobacterium;* internal transcribed spacer region (ITS) sequences were identified using BLAST in the NCBI database.
