# Supplementary figures and images for: Persistence of Supplemented Bifidobacterium longum subsp. infantis EVC001 in Breastfed Infants
Source: mSphere. 2017 Dec 6;2(6):e00501-17. doi: 10.1128/mSphere.00501-17 (PMC5717325; doi:10.1128/mSphere.00501-17)

Figure S1

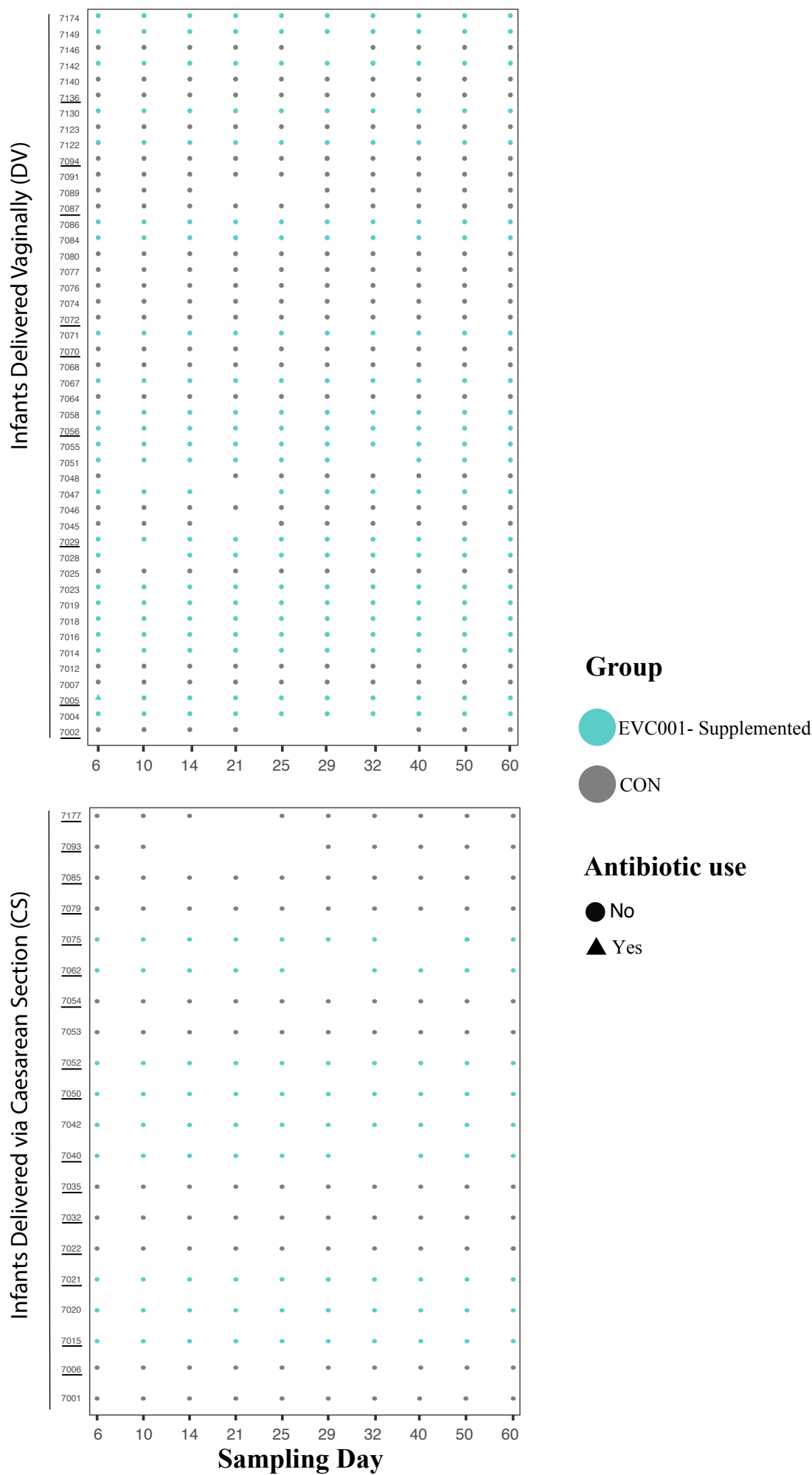

Supplement: FIG S1 [file sph006172417sf1.pdf]

Figure S2

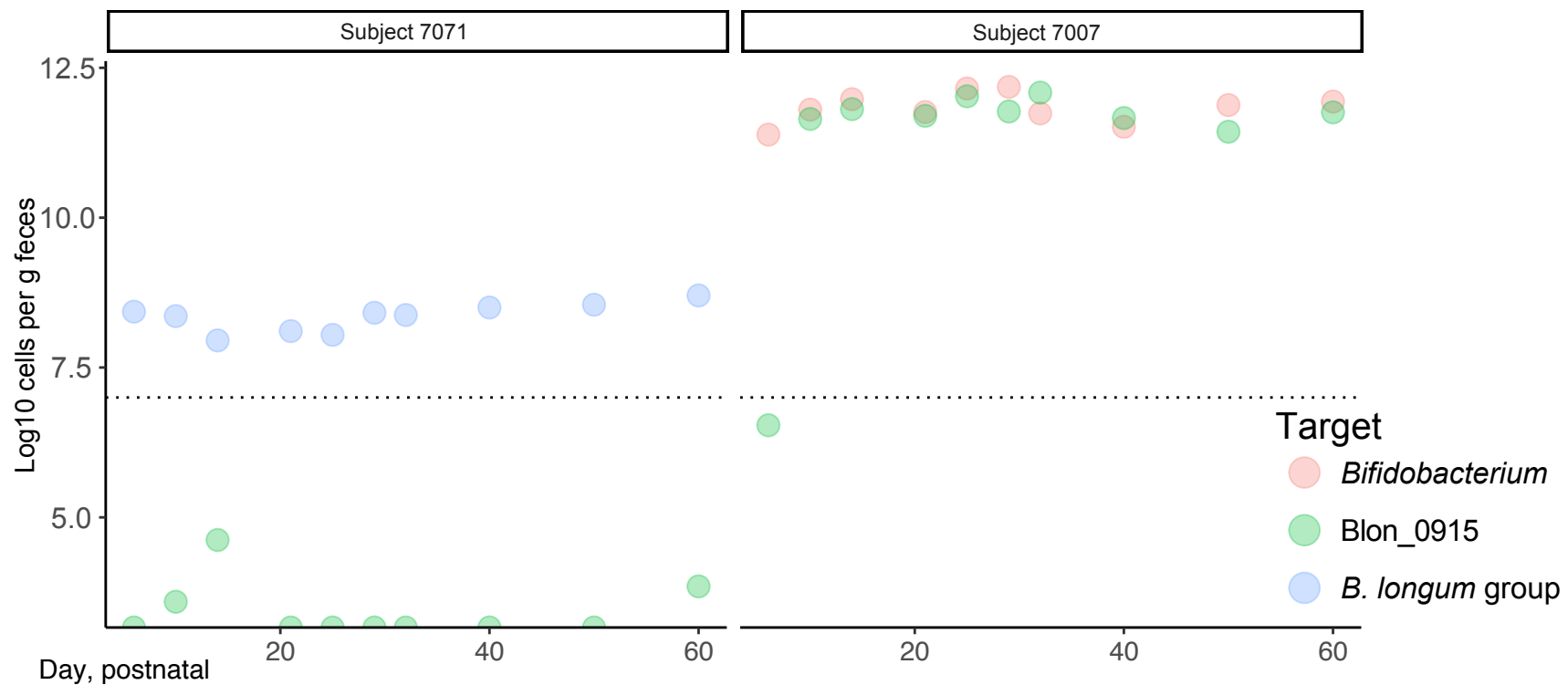

Supplement: FIG S2 [file sph006172417sf2.pdf]
