# Supplementary material for: Persistence of Supplemented Bifidobacterium longum subsp. infantis EVC001 in Breastfed Infants
Source: mSphere. 2017 Dec 6;2(6):e00501-17. doi: 10.1128/mSphere.00501-17 (PMC5717325; doi:10.1128/mSphere.00501-17)
Supplement: TABLE S2 [file sph006172417st4.pdf]

Table S2. Study population demographics[a]

Measure	Control, CON (SD)[b]	EVC001-fed (SD)	*P* value[c]
Group size	32	34	
Home births	1	2	NS ($P > 0.999$)
Hours in labor	18.77 (22.70)	12.88 (12.82)	NS ($P = 0.6656$)
Caesarean section births	9	11	NS ($P = 0.792$)
Mothers received antibiotics for labor	10	15	NS ($P = 0.3189$)
Gestational age (weeks)	40.05 (1.16)	39.53 (1.18)	NS ($P = 0.3769$)
Females	17	13	NS ($P = 0.3227$)
Birth weight (g)	3,590 (628)	3,458 (370)	NS ($P = 0.7564$)
Discharged weight (g)	3,382 (615)	3,256 (383)	NS ($P = 0.7564$)
Birth length (cm)	51 (3)	51 (2)	NS ($P = 0.9149$)
Infants received antibiotics prior to discharge	1	0	NS ($P = 0.4848$)
Birth complications (any)	6	0	$P = 0.0100$
Infants consumed formula before discharge	2	0	NS ($P = 0.2312$)
Maternal pre-pregnancy BMI	23.81 (3.20)	25.59 (3.61)	NS ($P = 0.2622$)
Pregnancy weight gain (kg)	15.37 (5.09)	15.00 (5.13)	NS ($P = 0.9149$)
Group B Streptococcus positive	5	9	NS ($P = 0.3708$)
Primiparous	25	14	$P = 0.0029$
Maternal age (years)	31.16 (3.42)	33.26 (4.52)	NS ($P = 0.2622$)
Non-secretor ($FUT2^{-/-}$)	5	6	NS ($P > 0.999$)

[a]Additional information has been reported previously (21).
[b]Values are presented as incidences or means with standard deviations in parentheses.
[c]Statistical tests were performed as Fisher's exact test or multiple *t*-tests with the Holm-Sidak correction as appropriate.
